# Using wearable data to detect depression severity across clinical and non-clinical samples

**DOI:** 10.1038/s41598-026-47177-3

**Published:** 2026-04-03

**Authors:** Miriam Ina Hehlmann, Rayyan Tutunji, Wolfgang Lutz, Carlotta L. Rieble, Ricarda K. K. Proppert, Fabienne Mink, Julian A. Rubel, Marieke Schreuder, Eiko I. Fried

**Affiliations:** 1 Osnabrück University, Lise-Meitner-Strasse 3, 49078 Osnabrück, Germany; 2 Leiden University, Leiden, Netherlands; 3 Trier University, Trier, Germany; 4 KU Leuven, Leuven, Belgium; 5 Tilburg University, Tilburg, Netherlands

**Keywords:** Depression severity detection, Passive sensing, Machine learning, Digital phenotyping, Multi-site data, Biomarkers, Diseases, Health care, Medical research

## Abstract

Early detection of depression is crucial, yet current assessment methods depend on self-report questionnaires and clinical interviews, which are resource-intensive. Wearable devices provide a scalable way to assess physiological and behavioral features, but their predictive value across clinical and non-clinical populations remains insufficiently established. Wearable-derived features were collected from a student sample (*n* = 187) and an outpatient sample (*n* = 95). Depressive symptoms were assessed using the Patient Health Questionnaire-9 (PHQ-9), and participants were categorized as screen-positive for depressed (≥ 10) or non-depressed (< 10). An elastic net regularized logistic regression model was used for classification, with performance evaluated in held-out test data. Sensitivity analyses controlled for age and bedtime, tested alternative PHQ-9 cutoffs, and comparisons to baseline models with and without wearable features. Across the combined sample (*n* = 282), the model achieved good discriminative performance (area under the curve = 0.82; accuracy = 79%). Sensitivity analyses revealed that sample was a strong predictor, but wearable-derived features still added incremental value. Minimum awake heart rate, variability in sleep duration, and maximum step count emerged as the strongest predictors. Wearable-derived features show promise for detecting depressive symptoms across clinical and non-clinical populations. Sample-specific factors should be considered in future research.

**Supplementary Information:**

The online version contains supplementary material available at 10.1038/s41598-026-47177-3.

## Introduction

Depression is one of the most pressing health-related issues, significantly impacting quality of life. It can cause severe and enduring functional impairments, reduce workplace productivity, increase the risk of suicidality, and is often considered a chronic disease^1–3^. Despite advances in treatments, their effectiveness has remained limited, with many individuals still experiencing depressive symptoms post-treatment^4,5^. As interventions in routine care are often initiated when symptoms are already well established, early and accurate detection may represent an important opportunity to prevent chronic courses and potentially improve treatment outcomes. Despite its importance, the assessment of depression largely relies on traditional methods such as clinical observations, patient history, and self-report questionnaire data^6^. These traditional assessment approaches, while valuable, present several challenges: they are time-consuming, often depend on potentially biased retrospective self-report^7^, and are further complicated by a growing shortage of mental health professionals^8^.

In recent years, wearable technologies such as smartwatches and fitness trackers have attracted attention as potential tools for unobtrusively detecting depression severity. They monitor physiological features such as heart rate, physical activity, and sleep patterns, which are closely related to core depressive symptoms including sleep disturbances and reduced energy or motivation^9^. These features are also among the most commonly used wearable-derived predictors in prior depression research, as documented in recent meta-analyses^10,11^. A recent meta-analysis by Abd-Alrazaq et al.^10^ and a systematic review by De Angel et al.^12^ support the potential of wearable data to detect depressive symptomatology. However, both reviews emphasize that the field is still at an early stage, with several challenges. First, half of the included studies relied on small samples (≤ 55 participants), calling for work in larger datasets. Second, current findings are limited in generalizability^10^, calling for studies in more diverse and representative populations. Third, while several recent large-scale studies^13,14^ include thousands of participants and advance the field methodologically, these samples are typically drawn from general populations without confirmed clinical diagnoses. It thus remains unclear whether wearable-based depression severity detection performs consistently across clinically diagnosed outpatients and non-clinical groups such as university students, and to our knowledge, no study has evaluated performance across both populations within the same analytic framework.

The current study aims to address these three challenges in the present work. To do so, we used two distinct datasets. Participants in both studies wore a Garmin device, which is a consumer-grade, wrist-worn wearable that passively records physiological and behavioral features such as heart rate, sleep, and daily activity. One dataset came from an outpatient sample consisting of individuals with a formal depression diagnosis, and the other from a student sample including mostly healthy controls. A single, mostly general-population sample would provide limited variability; by including these two complementary samples, we expected behavioral and physiological patterns to differ based on depression severity. The samples also differ in age and nationality, providing additional demographic variability. Together, the combined datasets allow a more comprehensive analysis by including larger, more diverse, and clinically relevant samples.

The present work is designed as a classification study, aiming to distinguish individuals who screen-positive for depression severity (≥ 10) from those who do not (< 10), based on a PHQ-9 cutoff. A classification framework was chosen because it aligns with the clinical purpose of screening and triage, indicating which individuals may warrant further evaluation. Thus, study has two main objectives: (1) to study the utility of wearable data for detecting depression severity, and (2) to identify the most predictive indicators for this purpose. By combining data from different countries and populations, and by using affordable, consumer-grade wearable technology, our research aims to advance understanding in this promising area and contribute to the development of scalable tools for depression severity detection.

## Results

After excluding participants with missing data, the final dataset comprised 282 participants. Among them, 129 were classified as depressed (47 WARN-D, 82 Trier) based on the predefined PHQ-9 cutoff (≥ 10), including 47 from the WARN-D sample and 82 from the Trier sample. On average, the Trier sample showed considerably higher depression severity scores (mean PHQ-9 = 15, *SD* = 4.67), than the WARN-D sample (*M* = 7.15, *SD* = 4.46). Table 1 presents the descriptive statistics (*M*,* SD*) of the predictor variables for depressed and non-depressed groups. Sample-specific descriptive statistics for each variable are provided in the supplemental material (Table 1).

**Table 1:** Descriptive Statistics of Predictor Variables Across Depressed and Non-Depressed Groups.

	Non-depressed (*n* = 153)	Depressed (*n* = 129)	
	M (SD)	M (SD)	*p*
Variable			
Steps			
Mean	6314 (2339)	6359 (2905)	0.89
Minimum	1333 (1494)	1536 (1569)	0.27
Maximum	12,968 (4650)	12,422 (5025)	0.35
SD	3355 (1305)	3182 (1341)	0.27
Sleep (in minutes)			
Mean	510 (45.40)	487 (61.30)	< 0.001
Minimum	373 (69.80)	339 (97.20)	< 0.001
Maximum	636 (73.70)	621 (92.40)	0.131
SD	74.8 (28.10)	84.6 (37.90)	0.016
Start time (in minutes from midnight)			
Mean	750 (363)	926 (372)	< 0.001
SD	503 (179)	356 (264)	< 0.001
Stop time (minutes from midnight)			
Mean	511 (83.8)	435 (121)	< 0.001
SD	76.8 (26.0)	87.7 (64)	0.073
Awake HR			
Mean	80.6 (7.75)	81.1 (8.10)	0.610
Minimum	61.3 (7.59)	60.4 (7.66)	0.300
Maximum	113 (12.3)	126 (20.90)	< 0.001
SD	12.1 (2.02)	13.4 (2.78)	< 0.001
Resting HR			
Mean	64.2 (8.04)	69.2 (9.32)	< 0.001
Minimum	55.7 (7.40)	58.7 (8.32)	0.002
Maximum	84.0 (9.61)	93.6 (15.80)	< 0.001
SD	5.72 (1.49)	7.21 (2.31)	< 0.001
Kurtosis	1.28 (1.90)	0.827 (1.77)	0.038
Skewness	1.06 (0.53)	0.929 (0.52)	0.043

*M* Mean, *SD* Standard deviation, *HR* Heart rate. Start time = beginning of sleeping period, Stop time = end of sleeping period.

The final elastic net model was selected via 10-fold cross-validation and used Ridge regularization (alpha = 0) with an optimal penalty parameter of lambda = 0.02. The model performed well in distinguishing participants above versus below the PHQ-9 cutoff^15^. On the independent test set, the model achieved an AUC of 0.82 (95% CI [0.75, 0.96]), accuracy of 0.79, and an F1-score of 0.82, with balanced sensitivity and specificity (see Table 2). The most influential predictors were the SD of sleep duration, minimum awake HR, and maximum step count; greater sleep variability, lower minimum awake HR, and fewer maximum daily steps were associated with depression severity. A full ranking of predictor importance, including variable importance, standardized coefficients, and odds ratios, is provided in Table 2 of the supplementary material.

To assess whether Garmin-derived features added predictive value beyond sample, we compared the elastic net model using Garmin features only (Garmin-only) to two subsequent models: a baseline logistic regression using sample only (Sample-only), and an elastic net model including sample plus Garmin features (Sample + Garmin). As shown in Table 2, the Sample-only model achieved high sensitivity but moderate specificity and a lower AUC and F1 score. The Sample + Garmin model improved overall performance, while the Garmin-only model achieved slightly lower AUC and accuracy but the highest F1 score and balanced sensitivity and specificity. Confusion matrices, provided in Table 3, summarize the number of correctly and incorrectly classified cases (true positives, true negatives, false positives, and false negatives) produced by the two elastic net models and the logistic regression model. The Garmin-only model produced the highest number of true positives and the lowest number of false negatives, with these values differing only slightly (by one participant) from the Sample + Garmin model, but notably more from the Sample-only model. However, the Garmin-only model also produced the highest number of false positives. Lastly, model performance exceeded chance, as confirmed by permutation testing (observed AUC = 0.82, permutation *p* = 0.002). Together, these findings indicate that while sample captures substantial predictive signal, Garmin-derived features also provide incremental discriminative value across models.

**Table 2:** Classification Performance across models: Sample-only, Sample + Garmin, and Garmin-only.

Model	Accuracy	Sensitivity	Specificity	AUC	F1 Score
Elastic Net Garmin-only	0.79	0.83	0.73	0.82	0.82
Sample-only	0.79	0.92	0.65	0.78	0.74
Elastic Net Sample + Garmin	0.81	0.89	0.70	0.85	0.77

Values show key performance metrics for each model. Sensitivity = proportion of correctly identified depressed cases; Specificity = proportion of correctly identified non-depressed cases; *AUC* area under curve.

**Table 3:** Confusion matrix for Sample-only, Sample + Garmin, and Garmin-only.

Model	Prediction	Reference 0	Reference 1
Elastic Net Garmin-only	0	40	10
	1	8	27
Sample-only	0	44	13
	1	4	24
Elastic Net Sample + Garmin	0	43	11
	1	5	26

The Sample-only model was estimated using logistic regression.

We conducted three additional sensitivity analyses to examine the robustness of the classification results. First, we re-estimated the models while controlling for age. Model performance decreased slightly, but the overall classification pattern remained stable (AUC = 0.71, Accuracy = 0.72, F1 Score = 0.78). In this model, the most influential predictors were variability in bedtime, mean end of sleeping period, and variability in resting HR. Second, we controlled for bedtime (start time in minutes from midnight). Here, model performance remained comparable to our Garmin-only model (AUC = 0.81, Accuracy = 0.74, F1 Score = 0.79). Here, the mean end of sleeping period emerged as the most influential feature, alongside variability and maximum levels of awake heart rate. For these two sensitivity analyses, overall model performance remained comparable, but the most influential predictors differed from those identified in the Garmin-only model. Third, we varied the PHQ-9 cutoff used for classification (thresholds of 9 and 11). These alternative cutoffs did not materially change model performance or predictor rankings (≥ 9 AUC = 0.75, Accuracy = 0.70, F1 Score = 0.70; ≥11 AUC = 0.79, Accuracy = 0.75, F1 Score = 0.73). Full results of these analyses are presented in supplementary material Tables 3 and 4.

## Discussion

This study examined the potential of consumer-grade wearable data to classify depression severity across two distinct datasets, one from an outpatient sample and another from a student sample. While models relied heavily on sample-related differences in the data for classification, the final elastic net model including Garmin data achieved the strongest classification performance. The most influential predictors for depression severity were greater variability (*SD*) in sleep duration, lower minimum awake HR, and fewer maximum daily steps. These findings contribute to the growing body of research suggesting that physiological and behavioral data from wearable devices may serve as valuable indicators for detecting depression severity.

The most influential predictors in our model spanned different domains rather than being confined to a single measure type, highlighting the potential advantage of integrating multiple data streams for depression severity classification. Greater variability in sleep duration was associated with higher depression severity, suggesting that participants with more irregular sleep patterns may experience more severe depressive symptoms. This aligns with the diagnostic criteria for depression, as sleep disturbances such as difficulties falling asleep or disrupted sleep are common symptoms^16^. Fewer maximum steps were associated with higher depression severity as well, suggesting that participants with lower daily activity tend to experience more severe depressive symptoms. This finding is consistent with previous research showing that individuals with depression tend to engage in lower levels of physical activity^11^. A lower minimum awake HR was associated with higher depression severity, suggesting that reduced physiological arousal or lower daytime activity may be characteristic of more severe symptoms. This finding could also reflect other factors, such as metabolic differences or reduced physiological reactivity, warranting further investigation. However, given the interdependencies among these features, future research should explore whether integrating diverse signals can meaningfully improve predictive performance beyond individual domains. Additionally, studies should explore the role of key predictors not only in detecting current depression severity but also in predicting future symptom trajectories.

Our findings align with a recent meta-analysis^10^ reporting high classification performance (accuracy = 0.89, sensitivity = 0.87, specificity = 0.93) for AI-based depression severity classification using Fitbit and Actiwatch data, and extend this literature to a new device (Garmin) and more heterogeneous populations, including students and outpatients. Our model showed slightly lower accuracy (79%) but comparable sensitivity (0.83). Unlike the mentioned AI models that favor detecting non-depressed individuals, it performed better at identifying depression severity. This could be due to our enriched sample, allowing for a broader range of depressive symptom severity and contributing to the model’s higher sensitivity. While our model showed strong performance, as in similar studies, caution is warranted due to potential overfitting.

Consistent with previous research, our study shows that passive sensing demonstrates some ability to detect depression severity, providing promising but not yet fully actionable results. Moving the field forward to fully leverage the potential of passive sensing will require several complementary strategies. First, our study represents an initial step by examining a diverse sample, made possible through collaboration across sites using the same device and similar assessment periods, similar to the implementation in infrastructures such as Remote Assessment of Disease and Relapse – Central Nervous System (RADAR-CNS^14,17^. Future progress will depend on fostering larger multi-site collaborations that employ harmonized assessment strategies, enabling not only larger datasets but also more diverse and representative data. Second, future research should adopt a multi-modal approach, combining wearable, smartphone sensors and EMA self-reports. Integrating physiological signals and self-reports with contextual information such as location or screen time could provide richer, more informative predictors. Finally, future studies should explore integrating passive sensing with EMA or clinical monitoring in structured workflows where sensor data supports but does not replace clinical judgment. Such approaches can help evaluate the real-world utility of predictive models and inform how they might be applied safely and effectively.

Several limitations should be considered when interpreting the findings. The performance of wearable devices may be limited by missing data. Many participants, particularly in the clinical population, were excluded because we required at least 50% coverage per feature. As shown in Supplemental Table 6, excluded outpatient participants were slightly younger and reported lower baseline depression severity, which may limit generalizability. Another limitation is that the two samples represent somewhat opposite ends of the depression spectrum; mostly healthy individuals and outpatients with a diagnosed depression. As a result, individuals with mild or subclinical depressive symptoms are underrepresented, which limits the study’s ability to support dimensional symptom monitoring or early detection. Consequently, the model’s predictions are likely driven by the contrast between active depressive episodes and healthy controls, and may be less precise for subclinical or emerging symptoms. Future work including participants across the full spectrum of depression severity will be needed to address this gap. Relatedly, the gap between cross-sectional classification and within-person prediction should be explicitly acknowledged. The present models are based on between-person differences and are designed to classify individuals relative to others, rather than to predict within-person change over time. Accordingly, within-person longitudinal modeling represents an important direction for future research. Moreover, PHQ-9 scores were collected before the wearable data, meaning the models capture concurrent associations rather than forecasting future symptom trajectories.

Regarding the student sample, the absence of clinically confirmed depression diagnoses reflects a common limitation in prior research that has relied solely on general-population samples. However, we extended this literature by additionally including an outpatient sample with clinically confirmed depression diagnoses. Nevertheless, wearable-derived features in the student sample may partially capture academic stress, which may influence the generalizability of these findings. Beyond this sample-specific limitation, the unbalanced distribution of depression status across samples limited our ability to train the model on one dataset and test it on the other. Future research should prioritize larger, more balanced multi-site datasets to reliably evaluate the contribution of passive sensing features within individual populations.

Measurement-related issues should also be noted. We used a binary PHQ-9 cutoff for classification, which reflects a commonly used threshold but does not capture the full spectrum of depressive severity. For the WARN-D dataset, but not the Trier data, a slightly adapted version of the PHQ was used from which the PHQ-9 was reconstructed, potentially introducing measurement non-equivalence and affecting comparability across sample. Furthermore, data collection for the student and outpatient samples occurred during partially non-overlapping calendar years. Although both datasets were collected continuously across all seasons, seasonal effects (e.g., winter vs. summer) were not explicitly modeled and therefore cannot be ruled out as a potential influence on wearable-derived physiological and behavioral patterns. Finally, while our study included participants from two samples representing both student and outpatient populations, generalizability remains limited. Several sociodemographic variables (e.g., gender identity, immigration history) were not collected, and the sample was relatively homogeneous (primarily German and Dutch participants). This is particularly relevant when using photoplethysmography-based HR measures, such as those employed by Garmin devices, as measurement accuracy and the extent of missing data may vary by skin tone, potentially limiting generalizability. Future studies should include broader and more diverse demographic information to enhance external validity.

This study advances research on wearable-based depression severity detection by leveraging consumer-grade smartwatches within a larger, more diverse sample. Our findings show both the promise and the complexity of using wearable data in detecting depression severity. While model performance may still be influenced by sample-related factors like demographic differences, our findings highlight the potential of wearable technology for mental health monitoring. Future cross-sample prediction efforts will be crucial to supporting its clinical translation.

## Methods

### Participants and procedure

The current study draws on data from two independent studies: the WARN-D study (Netherlands) and an outpatient study at Trier University (Germany). As the datasets were collected as part of separate projects, there are natural differences in study procedures, participant characteristics, and inclusion/exclusion criteria, which are described below.

#### WARN-D sample

The WARN-D study is a multi-cohort student study in the Netherlands, funded by the European Research Council (Horizon 2020, No. 949059), and with ethical approval by Leiden University (2021-09-06-E.I.FriedV2-3406). All procedures were performed in accordance with the relevant guidelines and regulations. Data for the WARN-D sample were collected continuously between April 2021 and October 2024, spanning all seasons. Participants in the WARN-D study were recruited via online and offline advertisements and partnerships with educational institutions, with interested individuals enrolling through an online survey. Here, the first two stages of WARN-D are relevant: After screening for inclusion criteria via an online screener, Stage 1 consisted of an online survey including depression measures that was filled out by all participants. In Stage 2, participants provided Garmin smartwatch data for three months. In the current study, we used only the first two weeks of wearable data to harmonize the assessment period with the outpatient sample, which was assessed for two weeks at the beginning of treatment. Exclusion criteria for WARN-D included moderate to severe depression severity (Patient Health Questionnaire-9 (PHQ-9)^18^ ≥14), current mania, thought disorders, primary substance use disorder, current or pending mental health treatment for any of the aforementioned, moderate to severe suicidal ideation, or distress from seeing daily calorie estimates. The WARN-D study focused on identifying early warning signs predicting depression onset; therefore, participants with moderate to severe depressive symptoms were excluded. Although the WARN-D study includes a large student sample, we selected a subset to match the Trier sample on age and reduce class imbalance between depressed and non-depressed participants (see Statistical Analyses for details). Further details on the WARN-D study are available Fried et al.^19^.

#### Trier sample

This study is embedded in the outpatient clinic of Trier University and funded by the German Research Foundation (LU 600/19 − 1), with ethical approval from Trier University (03.03.2022). All procedures were performed in accordance with the relevant guidelines and regulations. Data for the Trier sample were collected continuously between October 2019 and August 2024, spanning all seasons. Recruitment took place in the outpatient clinic of Trier University, where all individuals seeking treatment were invited to participate. As part of standard care, all patients completed pre-treatment assessments, including the PHQ-9, which were submitted at the initial interview. Study invitations were sent alongside initial interview invitations, during which inclusion and exclusion criteria were assessed. Exclusion criteria included current suicidality, psychosis, or mania. Exclusion criteria included current suicidality, psychosis, or mania. Eligible participants attended an introductory session and provided written informed consent for study participation and the use of their data for scientific purposes, but not for public sharing of individual-level data. Participants wore a Garmin fitness tracker for two weeks as part of their cognitive behavioral therapy (CBT), starting immediately after the diagnostic session (the second CBT session). During the diagnostic session, participants were assessed with Structured Clinical Interview for DSM-IV Axis I Disorders, and the diagnosis was confirmed by the treating clinician’s assessment, ensuring that all patients in the outpatient sample met criteria for a depressive disorder. Further details on study design and procedure are available in Hehlmann et al.^20^.

The final sample consisted of 282 participants (mean age = 26.95, *SD* = 9.83; 81% female). Table 4 provides an overview of the sociodemographic characteristics for WARN-D and Trier dataset. Please note that no data were collected on gender identity, and information on other diversity indicators such as socioeconomic background or immigration history is also limited.

**Table 4:** Sociodemographic Characteristics of Participant and Baseline Depression Severity.

	WARN-D (*n* = 187)	Trier (*n* = 95)
PHQ-9, Mean (SD)	7.15 (4.46)	15 (4.67)
Age, Mean (SD)	24.4 (7.77)	30 (11.10)
Sex, No. (%)		
Female	158 (84)	70 (74)
Male	29 (16)	23 (24)
Other	-	2 (2)
Nationality, No. (%)		
Dutch	111 (59)	-
German	13 (7)	86 (91)
Other	63 (34)	9 (9)
Family status, No. (%)		
Single	82 (44)	63 (66)
Married	5 (3)	15 (16)
Other	100 (53)	17 (18)
Educational Level, No. (%)		
Student	187 (100)	15 (16)
University degree	106 (57)	49 (51)
Other	-	31 (33)

*N* = 282. “Other” in Family status includes all relationship statuses beyond “single” and “married” (e.g., separated, divorced, widowed, living with a partner, or dating), which were harmonized across samples to ensure comparability.

### Measures

#### Depressive symptom severity

The 9-item PHQ-9 is a self-report measure, assessing depressive symptoms over the past two weeks on a 4-point scale (0 = not at all to 3 = nearly every day). Total scores range from 0 to 27, with higher scores indicating greater symptom severity. For the present study, baseline PHQ-9 scores were used for binary classification. Participants were classified as screen-positive for depression severity using a cutoff of ≥ 10^21^. In WARN-D, an adapted 15-item version of the PHQ-9 was used, from which PHQ-9 scores were reconstructed (see Table 5 in the supplemental material for more details).

#### Wearable data

Participants in both studies were instructed to wear a Garmin Vivosmart 4 smartwatch continuously, except for recharging. The device recorded HR every 15 s and provided daily measures such as step count, sleep duration, sleep start and end time (calculated as minutes from midnight). HR was assessed using photoplethysmography, which estimates pulse by detecting changes in light absorption due to blood flow. While Garmin devices provide these daily aggregated activity and sleep metrics, they do not offer continuous wear-time indicators or validated non-wear flags, which limits the ability to determine exact periods of non-wear. The wearable features examined in this study were selected to reflect largely device-agnostic constructs rather than Garmin-specific outputs. Accordingly, we focused on basic features that are available across many consumer-grade devices, while excluding proprietary composite metrics. Measurement characteristics and wearing modalities may vary across devices, so replication across platforms will be important to establish generalizability. Nevertheless, previous validation work has shown good agreement between Garmin heart rate measurements and electrocardiography^22^.

### Statistical analysis

Data processing and analysis were conducted in R version 4.4.0^23^, all scripts and supplementals are accessible online (https://osf.io/k37pw). To reduce class imbalance, we matched a subsample of the larger WARN-D dataset to the Trier sample by age using nearest-neighbor matching with the Mahalanobis distance in MatchIt (v4.7.0)^24^. This reduced but did not fully eliminate age differences.

Missing data in this study was handled by excluding participants (1) with missing PHQ-9 scores or (2) if wearable data coverage was below 50% for any given feature (see flowchart in the supplemental material, Fig. 1). With a 14-day monitoring period, we required ≥ 7 valid days to ensure stable person-level feature estimates. No imputation was performed, as participants with only 50% coverage would yield unreliable estimates; complete-case aggregation was used instead. To improve transparency, we additionally report sociodemographic characteristics and baseline depression severity of participants excluded according to the criteria in the supplemental material Table 6.

Several features were extracted: for step count and sleep duration, we calculated the mean (*M*), standard deviation (*SD*), minimum, and maximum values. To minimize the influence of date-related information that might bias the models, sleep start and end times were converted to times relative to midnight. HR was split into resting, measured during Garmin-recorded sleep periods, and awake. To control for outliers, the lowest and highest 5% of HR values were removed within person. For both resting and awake HR, we extracted the *M*, *SD*, minimum, and maximum values. Additionally, we calculated kurtosis and skewness for resting HR only, as resting HR is more stable over time compared with awake HR, which is subject to greater fluctuations due to activity, posture, and environmental factors. In total, 22 predictors were used for depression severity detection; all were z-transformed.

After merging the two datasets (WARN-D and Trier), participant IDs were randomly shuffled and assigned to a training (70%) or test (30%) set. All data from a given participant were assigned to only one set, ensuring independence between the training and test sets. Elastic net binary classification models were then fit using 10-fold cross-validation to predict the presence (1, PHQ ≥ 10) or absence (0, PHQ < 10) of depression severity from standardized wearable data. In this procedure, the training data were randomly split into 10 equally sized folds, with each fold used once as a validation set while the remaining nine folds were used to fit the model. Performance metrics were averaged across folds to identify the combination of alpha and lambda that achieved the best performance. The final model was then retrained on the full training set using these optimal hyperparameters and evaluated on the independent test set. We chose elastic net models to predict depression severity, as our dataset includes a large number of predictors and many wearable-data features are expected to be highly correlated. Elastic net handles both challenges effectively by combining variable selection and regularization^25^. Specifically, it combines L1 (lasso) and L2 (ridge) regularization: L1 encourages sparse models by shrinking some coefficients to zero, while L2 shrinks coefficients of correlated predictors toward each other. The balance between these penalties is controlled by the alpha parameter, and the overall strength of regularization is controlled by the lambda parameter.

The final model was then evaluated on the test set, with performance assessed using accuracy, AUC, and F1 score. Confusion matrices were used to summarize classification performance, including true positives, true negatives, false positives, and false negatives. Specificity was calculated as the proportion of participants below the PHQ-9 cutoff who were correctly classified by the model (true negatives). Variable importance was derived from the absolute magnitude of standardized model coefficients, reflecting each predictor’s contribution to the classification. Standardized coefficients and odds ratios are also reported to show the direction and magnitude of each predictor’s effect, noting that predictors with low importance can still have meaningful effects due to Elastic Net shrinkage and correlations among features. Finally, to confirm that model performance exceeded chance, permutation testing was conducted by shuffling outcome labels in the training set 500 times, and performance was evaluated on the original test set. This study was not preregistered.

### Sensitivity analyses

We conducted two sensitivity analyses to determine whether sample characteristics, such as study sample or age, would offer more accuracy than just the Garmin data. To account for potential sample-related influences, we fit a baseline logistic regression using sample as the sole predictor and an elastic net model including both sample and Garmin-derived features. The performance of these models was compared to the previously described elastic net model. Second, we used the combined dataset to address potential confounding by age and sleep timing. Specifically, age and time of going to bed were residualized within the training set only to prevent information leakage. The regression parameters obtained from the training data were then applied to the test set, and the elastic net model was re-estimated using the same procedure described above. Finally, we tested the robustness of our classification results by using alternative PHQ-9 cutoffs (≥ 9 and ≥ 11) to define depressed versus non-depressed individuals.

## Supplementary Information

Below is the link to the electronic supplementary material.

Supplementary Material 1

Supplementary Material 2

## Data Availability

The datasets analysed during the current study are subject to different availability conditions. The WARN-D dataset will be made publicly available in the WARN-D project repository (https://osf.io/frqdv/) once data collection, cleaning, and de-identification are complete. To make this article reproducible in the future, we share the exact participant IDs we used for this article in the supplementary materials. The Trier dataset on which study conclusions are based are not publicly available due to the sensitive nature of the information and the fact that participants did not provide explicit consent for data sharing. All R scripts and supplementary materials are publicly available at https://osf.io/k37pw.
