# Supplementary material for: Using wearable data to detect depression severity across clinical and non-clinical samples
Source: Sci Rep. 2026 Apr 3;16:11380. doi: 10.1038/s41598-026-47177-3 (PMC13049053; doi:10.1038/s41598-026-47177-3)
Supplement: Supplementary file 1 — Supplementary Material 1 [file 41598_2026_47177_MOESM1_ESM.pdf]

Using Wearable Data to Predict Depression in a Cross-Population Study

Supplementary Material

Credit Taxonomy

Funding:

Eiko I. Fried, Rayyan Tutunji, Ricarda K. K. Proppert, and Carlotta L. Rieble are supported by funding from the European Research Council (ERC) under the European Union's Horizon 2020 research and innovation program, Grant No. 949059.

Wolfgang Lutz and Fabienne Mink are supported by funding from the German Research Foundation (DFG), Grant No. LU 600/19-1 and Grant No. LU 660/20-1.

Acknowledgements:

We wholeheartedly thank all participants of WARN-D; all prior and current WARN-D team members; all members of the scientific advisory board of WARN-D; and all experts who participated in the Delphi study to inform our baseline measurement battery. We sincerely thank all participants of the Trier EMA study as well as all past and present team members for their valuable contributions to the project.

Competing Interests:

The authors have declared that no competing interests exists.

Author Contributions:

Miriam I. Hehlmann: Conceptualization; Data curation; Formal analysis; Methodology; Writing – original draft; Writing – review & editing.
Rayyan Tutunji: Conceptualization; Data curation; Investigation; Project administration; Validation; Formal analysis; Writing – review & editing.
Wolfgang Lutz: Conceptualization; Funding acquisition; Methodology; Project administration; Supervision; Writing – review & editing.
Carlotta L. Rieble: Data curation, Investigation; Project administration; Validation; Writing – review & editing.
Ricarda K. K. Proppert: Data curation; Investigation; Project administration; Validation; Writing – review & editing.
Fabienne Mink: Data curation; Investigation; Project administration; Validation; Writing – review & editing.
Julian A. Rubel: Methodology; Validation; Writing – review & editing.
Marieke Schreuder: Methodology; Validation; Writing – review & editing.
Eiko I. Fried: Conceptualization; Data curation; Funding acquisition; Methodology; Project administration; Supervision; Writing – review & editing.

Bluesky Accounts:

@miriamhehlmann.bsky.social, @rayyantutunji.bsky.social, @wlutzpsyres.bsky.social, @carlottarieble.bsky.social, @ricardaproppert.bsky.social, @fabiennemink.bsky.social, @julianrubel.bsky.social, @schreudermj.bsky.social, @eikofried.bsky.social

Data Availability:

We will make WARN-D data available on the WARN-D project hub (https://osf.io/frqdv/) when all data are collected, cleaned, and deidentified. To make this article reproducible in the future, we share the exact participant IDs we used for this article in the supplementary materials. The participants of the Trier EMA study did not provide written consent for their data to be

shared publicly. Due to the sensitive nature of the research, the supporting data from the Trier study is not available
