# Supplementary material for: Using wearable data to detect depression severity across clinical and non-clinical samples
Source: Sci Rep. 2026 Apr 3;16:11380. doi: 10.1038/s41598-026-47177-3 (PMC13049053; doi:10.1038/s41598-026-47177-3)
Supplement: Supplementary file 2 — Supplementary Material 2 [file 41598_2026_47177_MOESM2_ESM.pdf]

Using Wearable Data to Detect Depression Severity across Clinical and Non-Clinical Samples

Supplementary Material

Table 1

Descriptive Statistics of Predictor Variables Across Trier and WARN-D Datasets

	**WARN-D** (n = 187) *M (SD)*	**Trier** (n = 95) *M (SD)*	*p*
**Variable**			
**Steps**			
Mean	6070 (2558)	6855 (2642)	0.018
Minimum	1218 (1436)	1834 (1629)	0.002
Maximum	12746 (4872)	12662 (4753)	0.889
SD	3332 (1293)	3165 (1337)	0.328
**Sleep** (in minutes)			
Mean	511 (51.2)	477 (54.4)	< 0.001
Minimum	368 (72.4)	337 (103)	0.012
Maximum	642 (82.8)	604 (77.7)	< 0.001
SD	77.6 (29.8)	82.7 (39.2)	0.266
**Start time (in minutes from midnight)**			
Mean	698 (344)	1090 (295)	< 0.001
SD	527 (151)	257 (263)	< 0.001
**Stop Time (minutes from midnight)**			
Mean	529 (69.5)	372 (97.7)	< 0.001
SD	80.7 (27.5)	84.0 (72.5)	0.66
**Awake HR**			
Mean	80.8 (7.75)	80.9 (8.24)	0.95
Minimum	61.4 (7.66)	59.8 (7.47)	0.084
Maximum	112 (7.55)	133 (23.0)	< 0.001
SD	12.1 (1.87)	13.9 (3.04)	< 0.001
**Resting HR**			
Mean	63.7 (7.75)	71.9 (8.88)	< 0.001
Minimum	55.4 (7.30)	60.4 (8.20)	< 0.001
Maximum	83.1 (9.02)	98.8 (15.2)	< 0.001

SD	5.65 (1.55)	7.90 (2.08)	< 0.001
Kurtosis	1.20 (1.91)	0.82 (1.72)	0.085
Skewness	1.03 (0.54)	0.94 (0.51)	0.157

***Note.** M = Mean; SD = Standard deviation; HR = Heart rate.*

Table 2

Variable importance and standardized effect sizes for Garmin-derived features predicting depression

Variable	Contribution	Coefficient	Odds Ratio
**Steps**			
Mean	0.19	-0.052	0.949
Minimum	0.06	-0.013	0.988
Maximum	0.36	-0.096	0.909
SD	0.24	-0.003	0.997
**Sleep (in seconds)**			
Mean	0.24	-0.132	0.876
Minimum	0.05	-0.115	0.892
Maximum	0.09	-0.014	0.986
SD	0.44	0.169	1.184
**Start time (in seconds)**			
Mean	0.004	0.087	1.091
SD	0.22	-0.202	0.817
**Stop time (in seconds)**			
Mean	0.33	-0.215	0.806
SD	0.02	0.037	1.038
**Awake HR**			
Mean	0.36	-0.145	0.865
Minimum	0.39	-0.158	0.854
Maximum	0.35	0.189	1.209
SD	0.23	0.131	1.140
**Resting HR**			
Mean	0.19	0.106	1.112
Minimum	0.24	0.078	1.081
Maximum	0.30	0.137	1.147
SD	0.06	0.108	1.114
Kurtosis	0.18	-0.098	0.906
Skewness	0.11	-0.085	0.918

***Note.** M = Mean; SD = Standard deviation; HR = Heart rate. We applied an Elastic Net to predict depression from Garmin wearable features. Variable importance was calculated*

using the caret varImp() function, representing the relative contribution of each predictor to the model's predictive accuracy across cross-validation folds. Standardized coefficients and corresponding odds ratios indicate the direction and magnitude of the effect for each predictor. Because Elastic Net shrinks coefficients and accounts for correlations among predictors, variables with low contribution can still have non-negligible effect sizes. This table provides both predictive importance and standardized effects for transparency and interpretability.

Table 3

Sensitivity Analysis of Age and Bedtime Control on Prediction Performance

	Prediction of Depression controlled for	
	Age	Bedtime
**Accuracy**	0.72	0.74
**AUC**	0.71	0.81
**F1 Score**	0.78	0.79

Note. *AUC = Area under the curve*

Table 4

Sensitivity Analysis of Patient Health Questionnaire-9 Cutoff Variation

	Prediction of Depression for PHQ-9 cutoff	
	≥9	≥11
**Accuracy**	0.7	0.71
**AUC**	0.75	0.75
**F1 Score**	0.7	0.73

Note. *PHQ-9 = Patient Health Questionnaire-9, AUC = Area under the curve.*

Adapted PHQ-9 in the WARN-D Study

Table 1 shows the adapted PHQ-9 (Kroenke et al., 2001) asked in the baseline survey (Stage 1) in the WARN-D study. For WARN-D, any items from the original PHQ-9 that aggregate different symptoms were disaggregated. Specifically, the disaggregated symptoms were: sad mood and hopelessness (originally PHQ-9 item 2); insomnia and hypersomnia (originally PHQ-9 item 3), appetite loss and overeating (originally PHQ-9 item 5), and motor agitation and retardation (originally PHQ-9 item 8). Disaggregating these items allows for a more differentiated understanding of people's problems. Additionally, a question about the commonly experienced symptom libido loss was added[1].

[1] Of note, in the EMA period (Stage 2) of WARN-D, an item asking about the commonly experienced symptom of irritability was also added to the adapted PHQ-9. In the baseline (Stage 1) and follow-ups (Stage 3), this was not done, as a corresponding item from the GAD-7 (Spitzer et al., 2006) was available ("gad6_annoyed").

From the adapted version, a person's original PHQ-9 sum score can be deduced. The additional symptom (libido loss) is not used. The other items are summed; for each original PHQ-9 item, the maximum value of the two items it was disaggregated into is used.

Table 5

Adapted PHQ-9 in the WARN-D Baseline Survey

Construct	Variable	Question
Anhedonia	phq1_anhedo	Little interest or pleasure in doing things
Sad Mood	phq2_sad	Feeling down or depressed
Hopelessness	phq2_hopeless	Feeling hopeless
Insomnia	phq3_insomnia	Trouble falling asleep or staying asleep
Hypersomnia	phq3_hypersom	Sleeping too much
Tiredness	phq4_tired	Feeling tired or having little energy
Decreased Appetite	phq5_appet_loss	Poor appetite
Overeating	phq5_overeat	Overeating
Worthlessness	phq6_worthless	Feeling bad about myself – or that I'm a failure or have let myself or my family down
Concentration Problems	phq7_concentr	Trouble concentrating on things, such as reading or watching television
Motor Retardation	phq8_retar	Moving or speaking so slowly that other people could have noticed
Motor Agitation	phq8_agitate	Being so fidgety or restless that I have been moving around a lot more than usual
Irritability	phq11_irrit	Feeling irritable
Decreased Libido	phq12_libido	Little interest in sex
Suicidal Ideation	phq9_suicide	Thoughts that I would be better off dead or of hurting myself in some way
Pseudo PHQ-9 sum score	pseudo_phq9_sum	Calculated based on this formula:<br><br>sum(phq1_anhedo, max(phq2_sad, phq2_hopeless), max(phq3_insomnia, phq3_hypersom), phq4_tired, max(phq5_ appet_loss, phq5_overeat), phq6_worthless, phq7_concentr, max(phq8_retar, phq8_agitate), phq9_suicide)

Note. *These items were presented with the overarching question "Over the past 2 weeks, how often have you been bothered by the following problems?"; all variables were rated on a four-point scale ranging from 0 to 4 with the answer options: "Not at all", "Several days", "More than half the days", and "Nearly every day". The pseudo PHQ-9 sum score ranges from 0 to 27, with higher scores indicating higher depression severity.*

Figure 1

Flowchart of Participant Exclusions for the Final Sample

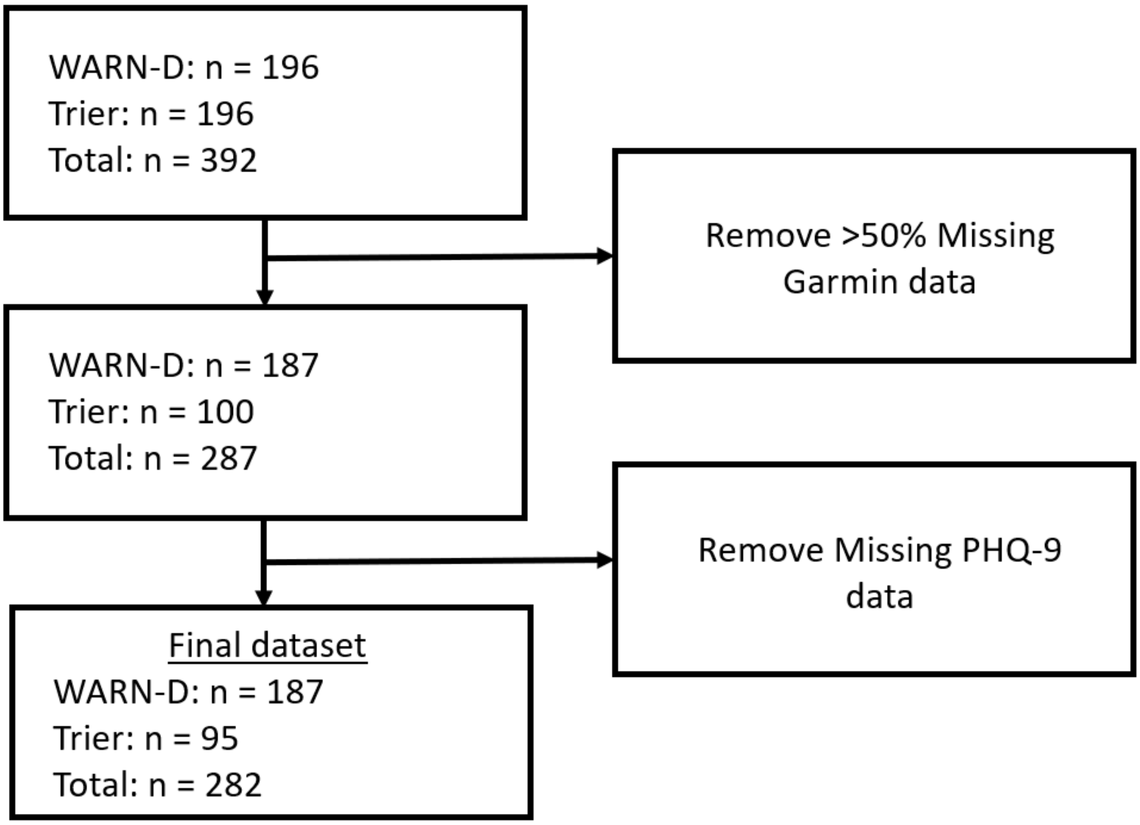

Note. *Missing data in this study was handled by excluding participants 1) with missing PHQ-9 scores or 2) if wearable data coverage was below 50% for any given feature.*

Table 6

Sociodemographic Characteristics and Baseline Depression Severity of Dropout Participant

	**WARN-D** (n = 9)	**Trier** (n = 101)
PHQ-9, Mean (SD)	7.83 (4.95)	13.35 (5.35)
Age, Mean (SD)	20.83 (1.17)	32.25 (12.04)
Sex, No. (%)		
Female	6 (66.67)	70 (69.31)
Male	-	27 (26.73)
Other	-	4 (3.96)
Nationality, No. (%)		
Dutch	4 (44.44)	-
German	2 (22.22)	94 (93.07)
Other	3 (33.33)	7 (6.93)
Family status, No. (%)		
Single	3 (33.33)	67 (66.34)

Married	1 (11.11)	17 (16.83)
Other	5 (55.56)	17 (16.83)
Educational Level, No. (%)		
Student	9 (100)	19 (18.81)
University degree	-	35 (34.65)
Other	-	47 (46.53)

Note. *N = 110. "Other" in Family status includes all relationship statuses beyond "single" and "married" (e.g., separated, divorced, widowed, living with a partner, or dating), which were harmonized across samples to ensure comparability.*
